# Bulbar Onset Amyotrophic Lateral Sclerosis in an African American Older Adult

**DOI:** 10.31486/toj.23.0045

**Published:** 2023

**Authors:** Arielle Kasindi, Kathy Jo Carstarphen

**Affiliations:** ^1^ The University of Queensland Medical School, Ochsner Clinical School, New Orleans, LA; ^2^ Department of Internal Medicine, Ochsner Clinic Foundation, New Orleans, LA

**Keywords:** *Amyotrophic lateral sclerosis*, *delayed diagnosis*, *dysarthria*, *lower urinary tract symptoms*, *motor neuron disease*, *religion*

## Abstract

**Background:** Amyotrophic lateral sclerosis (ALS), a fatal neuromuscular disease, affects the motor tracts and anterior horn cells of the spinal cord, causing both upper and lower motor neuron dysfunction. ALS typically involves progressive peripheral weakness and mobility issues.

**Case Report:** An African American male in his early 70s presented to his primary care provider (PCP) with bulbar weakness and urinary tract symptoms. At presentation, and even later in the disease course, he ambulated well and did not report any limb issues. After some months of worsening symptoms of dyspnea, dysarthria, dysphagia, and urinary incontinence, a diagnosis of ALS was made via collaborative work between the PCP, a medical student, and various medical specialists including a neurosurgeon and neurologist. Because of the absence of limb abnormalities, the patient had difficulty accepting a diagnosis of ALS, thus delaying treatment onset.

**Conclusion:** Clinicians must consider the patient's presentation holistically so that they do not miss insidious, complex, or unique presentations. ALS can present with bulbar symptoms early in the disease course with no upper motor neuron/lower motor neuron involvement. Older adult African American males can present with ALS. Mistrust of health care systems and resistance to science based on religious beliefs can impact patient acceptance of diagnoses and engagement in treatments. Having a long-term relationship with a PCP who also represents the patient's community can influence patient willingness to accept diagnoses, especially those that are life-limiting.

## INTRODUCTION

Amyotrophic lateral sclerosis (ALS), commonly known as Lou Gehrig disease, is a progressive neuromuscular disease with high morbidity and mortality.^1^ A study by Bianchi et al reported a 5-year survival rate of 24% and a median survival of 2.4 years after diagnosis.^2^ ALS is commonly diagnosed in patients between 50 and 68 years of age, with an average age of onset/diagnosis of 55 years.^3^ White males are more commonly affected than any other demographic.^4^ Patients typically present because of motor dysfunction in the periphery (limb weakness, gait instabilities, mobility issues), and signs are often progressive over time.^5^ ALS affects the anterior horn cells of the spinal cord, the area of the spinal cord in which motor neural tracts run from the primary motor cortex in the brain to the neuromuscular junctions in the peripheral muscles. In ALS, these tracts are affected, thus causing upper motor neuron and lower motor neuron dysfunctions. Having mixed signs of both is the classic presentation of ALS.^5^

We present the case of a patient with delayed diagnosis of progressive bulbar onset ALS.

## CASE REPORT

An African American male in his early 70s initially presented to his primary care physician (PCP) with mild voice changes. His medical history was relevant for obstructive sleep apnea, benign prostatic hyperplasia, hypertension, and type 2 diabetes mellitus. The patient was the pastor of a large church, and his dysarthria progressively worsened during the next few months until his speech became largely unintelligible, resulting in his inability to lead the church choir in song or deliver a sermon. The patient also reported that he was experiencing dysphagia with solids. These symptoms became life-altering for the patient; he could not carry out his activities of daily living or efficiently perform his job.

The patient concurrently experienced dyspnea and urinary incontinence, initially thought to be attributable to his previously diagnosed obstructive sleep apnea and benign prostatic hyperplasia. Dyspnea was principally brought on with exertion, and the patient noted occasional wheezing. The patient's dyspnea became so concerning that he presented to the emergency department on multiple occasions.

Initially, the patient had significant issues with micturition and often experienced dribbling, straining, and nocturia that clinically correlated to his benign prostatic hyperplasia diagnosis. He took tamsulosin (Flomax) for these symptoms. However, his urinary urgency progressively worsened to urinary incontinence. He took a change of clothes wherever he went, anticipating incontinence. The urinary incontinence became increasingly distressing for the patient. His PCP later determined, after consulting a urology specialist, that these symptoms were not attributable to benign prostatic hyperplasia. Frequent and severe urinary incontinence is not commonly associated with benign prostatic hyperplasia but typically has a neurologic etiology.

The patient's dyspnea, dysarthria, dysphagia, and urinary incontinence were evaluated independently by several subspecialists. The patient received extensive workups, but results were negative for pathologic processes.

Stroke and cerebrovascular accident were initially the principal differential diagnoses. Because the patient's initial chief complaints were voice changes, dysphagia, and dysarthria, all signs of stroke,^6^ he was referred to vascular neurology. However, magnetic resonance imaging and computed tomography (CT) imaging were negative, ruling out a neurovascular cause of the symptoms.

The patient stated that he felt his eyelids drooping while watching television and endorsed worsening of speech symptoms if he spoke for long periods. Given the progressive worsening of muscles with repeated use,^7^ he was assessed for myasthenia gravis. However, serologic testing was negative.

In addition to the rapidly progressive voice changes, the patient frequently complained of dyspnea. Because of the patient's distant history of smoking, lung neoplasm was suspected. Additionally, paraneoplastic syndrome, such as Lambert-Eaton syndrome, could have explained the patient's neuromuscular issues.^8^ However, no lung mass was visualized on CT, the patient had no other signs or symptoms related to lung cancer, and all other pulmonary testing was normal. Lambert-Eaton syndrome would not fully explain all the issues the patient was experiencing.

Congestive heart failure typically presents with dyspnea, often orthopnea and paroxysmal nocturnal dyspnea.^9^ Although the patient reported normal breathing while he was lying flat, he experienced difficulties with obstructive sleep apnea. He had several risk factors for cardiovascular disease, so he was assessed for congestive heart failure and/or myocardial infarction. All cardiovascular examinations were negative.

In March 2021, after approximately 6 months of worsening symptoms, the patient's speech was so severely slurred and dysarthric that he was difficult to understand. He reported choking on his own saliva and experiencing severe dysphagia. A full neurologic examination showed reduced reflexes and bilateral upper extremity fasciculations. Otherwise, his neurologic examination was normal. The patient's PCP, a medical student, and a medical resident pieced together the components of the patient's presentation and specialist workups to arrive at the differential diagnosis of motor neuron disease. He was referred to a motor neurologist for further evaluation.

In April 2021, 7 months after the initial onset of symptoms of dysarthria, the patient was evaluated by a neurologist who specialized in neuromuscular conditions. A thorough examination in the neuromuscular clinic identified fasciculations in the left triceps muscle, left abductor hallucis muscle, and right abductor pollicis brevis muscle without noticed weakness. On ambulation, the patient's gait was slow and wide-based, and he was unable to walk on his toes. Table 1 presents the reflex and muscle strength findings from the examination. Slight drooling and right eye ptosis were also noted. The patient's speech was largely unintelligible with significant spastic dysarthria. The patient could not articulate his words, his voice was weak and nasal sounding, and his speech was slowed.

**Table 1.** Reflex and Muscle Strength Findings

Reflex/Muscle Group	Left	Right
Reflex^a^		
Biceps	2+	2+
Brachioradialis	3+	3+
Triceps	2+	2+
Patellar	2+	0
Ankle	0	0
Plantar	Up	Up
Muscle group^b^		
Interossei	5	4
Abductor pollicis brevis	5	4
Toe extension	4	5
Toe flexion	4	5

^a^Reflex findings: 0, absent reflex; 1+, trace; 2+, normal; 3+, brisk; 4+, nonsustained clonus; 5+, sustained clonus; up, pathological (past 2 years of age).

^b^Muscle strength findings: 5, normal strength; 4, movement against some resistance; 3, movement against gravity but not resistance; 2, limb movement but not against gravity; 1, visible muscle contraction no movement; 0, no visible muscle contraction.

ALS is largely a clinical diagnosis or a diagnosis of exclusion. Therefore, to confidently diagnose ALS, other disease states must first be ruled out. The clinical suspicion of ALS increased as the patient continued to lose motor function of various muscular groups. The detailed neuromuscular workup, with specific attention paid to subtle upper and lower motor neuron signs, supported the likelihood that the patient had ALS. Considering the negative findings from other investigations and the progressive nature of the patient's symptoms, ALS became the principal differential diagnosis.

No treatments for ALS are curative or can reverse the disease state. However, one pharmaceutical option—riluzole—has been shown to reduce disease progression.^10^ Riluzole works as an antagonist against glutamate release in the central nervous system and is one of the only US Food and Drug Administration–approved medications for the management of ALS. The patient was prescribed this therapy and enrolled at the Ochsner ALS Center, a multidisciplinary program that has the Certified Treatment Center of Excellence designation from the ALS Association.^11^

The patient initially rejected the ALS diagnosis. He had poor trust in the medical system and did not agree with the majority of what his medical providers told him regarding his diagnosis. He relied heavily on his religious background for guidance. As numerous providers pointed to the same diagnosis, he preferred to “pray for a miracle” and “rebuke the diagnosis.” The patient had a strong rapport with his PCP, who represented his community, and he trusted her medical decision-making. Regardless, he asked for a second opinion from a trusted neurosurgeon who had performed a surgery on his son that he described as “a miracle.” The patient felt that going to the Ochsner ALS Center would be an acceptance of the ALS diagnosis and he was extremely hesitant to do so. Consequently, several months elapsed before he presented to the ALS center after his diagnosis. However, once he began the program, the patient reported improvement of his symptoms. The ALS center focuses on multiple aspects of the disease, providing both physical therapy and respiratory therapy, and offers education, nutrition, therapy, support groups, familial assistance, and other interventions. Patients enrolled in the program receive help from speech therapy to assist with vocal and swallowing difficulties—the principal concerns of our patient. His vocal and bladder control and his ambulation improved. The patient learned ways to improve bladder control and received respiratory assistance. He agreed to take riluzole and was compliant with all recommended therapies.

However, 1 year after initial presentation, the patient declined rapidly and died while enrolled in hospice.

## DISCUSSION

The patient's presentation and disease course were unique and caused difficulty diagnosing his condition. He did not present with the classic mixed upper motor neuron/lower motor neuron ALS signs and progressive mobility issues. Instead, his chief complaints at the initial presentation were dysarthria and dysphagia, indicating bulbar involvement.^12^ Additionally, the patient reported increasing dyspnea and urinary incontinence. Although bulbar onset ALS accounts for approximately 30% of ALS presentations/subtypes,^13^ respiratory muscle involvement is typically a late disease complication and not generally present at disease onset.^14^ Additionally, approximately 26.3% of ALS patients report urgency urinary incontinence.^15^ This constellation of symptoms, the lack of obvious limb involvement, and the patient's age and ethnicity represent a rare presentation for ALS.^16^

As outlined in Table 2, 7 months elapsed from presentation to diagnosis. If the patient had had mobility issues or obvious mixed upper motor neuron/lower motor neuron signs, perhaps his diagnosis would have been made sooner. Instead, his symptoms were evaluated separately rather than as signs of the same pathologic process. The patient was sent to several specialists with delays in assessing the symptoms globally and inferring a link between them. Each specialty team did an extensive workup, resulting in unnecessary use of medical resources (an issue of distributive justice), increased cost to the patient, and a heightened burden on an already overextended health system.

**Table 2.** Timeline of the Patient's Symptoms and Clinical Course

September 2020	November 2020	December 2020	February 2021	March 2021	April 2021	March 2022
First reports of voice changes	Reports slurred speech and inability to give sermon	Workups negative for acute stroke, myasthenia gravis, heart failure, and dysphagia	Starts speech therapy, first reports of urinary incontinence	Speech and urinary incontinence symptoms worsen, concern for paraneoplastic process or motor neuron disease	Diagnosed with ALS	Death

ALS, amyotrophic lateral sclerosis.

## CONCLUSION

This case outlines the clinical significance of identifying a bulbar onset of ALS, particularly in patients of nondominant demographics. The importance of interdisciplinary communication is demonstrated in this case; the PCP coordinated care among the multiple teams and provided improved health outcomes. Additionally, this case highlights the importance of establishing strong patient rapport and considering sociologic and cultural influences on patients’ health literacy, capacity, and decision-making.

## Notes

*This article meets the Accreditation Council for Graduate Medical Education and the American Board of Medical Specialties Maintenance of Certification competencies for Patient Care and Medical Knowledge.*
